# The effects of iron oxide nanoparticles on antioxidant capacity and response to oxidative stress in Mozambique tilapia (*Oreochromis mossambicus*, Peters 1852)

**DOI:** 10.2478/aiht-2024-75-3826

**Published:** 2024-06-29

**Authors:** Puthan Variyam Vidya Balakrishnan, Goran Gajski, Kumari Chidambaran Chitra

**Affiliations:** University of Calicut, Department of Zoology, Endocrinology and Toxicology Laboratory, Malappuram, India; Institute for Medical Research and Occupational Health, Division of Toxicology, Zagreb, Croatia

**Keywords:** antioxidant enzymes, bioaccumulation, environmental health, oxidative stress, toxicity, antioksidacijski enzimi, bioakumulacija, oksidacijski stres, toksičnost, zdravlje okoliša

## Abstract

Recent research has raised concern about the biocompatibility of iron oxide nanoparticles (IONPs), as they have been reported to induce oxidative stress and inflammatory responses, whilst prolonged exposure to high IONP concentrations may lead to cyto-/genotoxicity. Besides, there is concern about its environmental impact. The aim of our study was to investigate the effects of IONPs on the antioxidant defence system in freshwater fish Mozambique tilapia (*Oreochromis mossambicus,* Peters 1852). The fish were exposed to IONP concentration of 15 mg/L over 1, 3, 4, 15, 30, and 60 days and the findings compared to a control, unexposed group. In addition, we followed up the fish for 60 days after exposure had stopped to estimate the stability of oxidative stress induced by IONPs. Exposure affected the activity of antioxidant and marker enzymes and increased the levels of hydrogen peroxide and lipid peroxidation in the gill, liver, and brain tissues of the fish. Even after 60 days of depuration, adverse effects remained, indicating long-term nanotoxicity. Moreover, IONPs accumulated in the gill, liver, and brain tissues. Our findings underscore the potential health risks posed to non-target organisms in the environment, and it is imperative to establish appropriate guidelines for safe handling and disposal of IONPs to protect the aquatic environment.

## Sažetak

Nedavna istraživanja izazvala su zabrinutost oko biokompatibilnosti nanočestica željezova oksida (engl. *iron oxide nanoparticles* – IONP), nakon što je utvrđeno da izazivaju oksidacijski stres i upalni odgovor, a produljena izloženost visokim koncentracijama IONP-a može dovesti do cito-/genotoksičnosti. Osim toga, postoji i zabrinutost u pogledu njihova utjecaja na okoliš. Cilj ovog istraživanja bio je proučiti djelovanje IONP-a na antioksidacijski obrambeni sustav slatkovodne ribe mozambičke tilapije (*Oreochromis mossambicus*, Peters 1852). Ribe su bile izložene koncentraciji IONP-a od 15 mg/L tijekom 1, 3, 4, 15, 30 i 60 dana, a usporedno su praćene i jedinke kontrolne, neizložene skupine. Nadalje, praćenje je nastavljeno tijekom 60 dana nakon prestanka izloženosti kako bismo procijenili stabilnost oksidacijskoga stresa izazvanoga IONP-om. Izloženost je utjecala na aktivnost antioksidacijskih i markerskih enzima te povećala razine vodikova peroksida i lipidne peroksidacije u tkivu ribljih škrga, jetre i mozga. Čak i nakon 60 dana „čišćenja“ zaostali su štetni učinci, koji upozoravaju na nepovratnu nanotoksičnost. Štoviše, IONP se akumulirao u tkivu škrga, jetre i mozga. Naša otkrića naglašavaju potencijalne zdravstvene rizike za neciljane organizme u okolišu, te je nužno uspostaviti odgovarajuće smjernice za sigurno rukovanje i odlaganje IONP-a kako bi se zaštitio vodeni okoliš.

Iron oxide nanoparticles (IONPs) are usually composed of magnetite (Fe_3_O_4_) and maghemite (γ-Fe_2_O_3_). They are highly adsorptive due to small size and large surface area-to-volume ratio and have magnetic properties, which make them useful in numerous applications such as biomedicine, environmental remediation, magnetic storage and recording media, catalysis, and magnetic fluids. They can be synthesised using various methods, including co-precipitation, thermal decomposition, sol-gel synthesis, and microemulsion techniques, which can tune IONP properties to specific application by changing their size, shape, surface chemistry, and crystalline structure during synthesis. Although they are characterised by low toxicity and cost, they may still be toxic and entail environmental risks, especially with biomedical and environmental applications (1,2,3,4,5,6,7,8,9).

Such concerns are well grounded due to their widespread use and potential release into the environment. IONPs can easily reach air, water, and soil thanks to small size and high surface area-to-volume ratio, which enhance their mobility and persistence in the environment, leading to potential long-term exposure and accumulation in various environmental compartments. One of the key environmental hazards associated with IONPs is their toxicity to aquatic organisms, including fish, algae, and other aquatic life, with adverse effects including impaired growth, reproduction, and survival of organisms exposed to high concentrations. Besides, they have the potential to bioaccumulate in aquatic organisms through ingestion or absorption and to biomagnify, which means that IONP concentrations increase as they move up the food chain, posing higher risks to higher trophic levels, including humans (4, 10,11,12,13,14,15). Moreover, IONPs may interact with other pollutants or environmental stressors, leading to synergistic or antagonistic effects on ecosystems (10, 16,17,18).

The profound effect of IONPs on haematology, ion regulation, and gill Na/K ATPase activity has been demonstrated in the Indian major carp (*Labeo rohita)* (19), while other studies have reported oxidative stress and genetic damage in Japanese medaka embryos (*Oryzias latipes*) (20), zebrafish (*Danio rerio*) (21), and rainbow trout (*Oncorhynchus mykiss*) (22).

Fish tissues contain a large amount of polyunsaturated fatty acids, which are highly vulnerable to oxidative stress, followed by tissue damage. Oxidative balance in cells or tissues is maintained by a well-equipped prooxidant/antioxidant defence system that scavenges free radicals, yet oxidative stress varies between tissues and may affect them differently.

Despite considerable research of IONP toxicity in fish, there are still knowledge gaps concerning their long-term adverse effects. Therefore, one aim of this study was to investigate the effects of IONPs on antioxidant defence and susceptibility to oxidative stress in the gill, liver, and brain tissues, and whether the nanoparticles get accumulated inside these tissues of the juvenile Mozambique tilapia (*Oreochromis mossambicus*, Peters 1852). Our second aim was to estimate long-term (60 day) presence of oxidative stress induced by IONPs, once the exposure has stopped, and consequent tissue damage using specific marker enzymes.

## MATERIALS AND METHODS

### Test organism and exposure conditions

For this study we used the juvenile Mozambique tilapia (*Oreochromis mossambicus,* Peters 1852), weighing 6.0±1.5 g and measuring 6.5±1.0 cm in average, obtained from the Safa Aquarium (Kozhikode, India). During the two-week acclimatisation period, a total of 160 fish were housed in a 180 L cement tank under a 12-hour light/dark cycle, where they were provided with a continuous supply of dechlorinated tap water. The fish were fed standard commercial pellets (Taiyo, Uthukkottai, India) on a daily basis, which contained the appropriate amount of nutrients including 41 % crude protein, 6 % crude lipids, 7 % crude fibre, 10 % carbohydrate, 15 % ash, <8.5 % phosphorus, <1.5 % water, and trace amounts of vitamins and minerals. Their health status was monitored throughout the experiment following the guidelines set forth by the Indian Committee for the Purpose of Control and Supervision of Experiments on Animals (CPCSEA) (23).

Optimal physico-chemical characteristics of the tap water were maintained and monitored in line with the standard methods published by the American Public Health Association (APHA) (24) and the OECD guideline on acidity and alkalinity (25) as follows: temperature 28±2 °C, pH 7.0±0.5, dissolved oxygen 8.6±0.6 mg/L, water hardness 48 mg/L calcium carbonate, total organic carbon 0.04 mg/L, non-ionised ammonia 0.01 mg/L, nitrate 10 mg/L, chlorine 4 mg/L, metallic impurities <1 mg/L, and chemical oxygen demand 3 mg/L. Water analyses were done to ensure that water did not affect the experimental outcomes.

### Characterisation of the test chemical

Preparation and characterisation of IONPs used in this study has been described in detail in our previous publications (26, 27). Briefly, IONPs (Fe_3_O_4_-NPs; Cat. No. 637106) were obtained from Sigma (Darmstadt, Germany). X-ray diffraction (XRD; Rigaku Miniflex, Tokyo, Japan) and transmission electron microscopy (TEM; Jeol/JEM, Tokyo, Japan) were performed to confirm the size and purity of IONPs. IONPs were suspended in double-distilled water and sonicated in an ultrasonic bath (GT Sonic, Guangdong, China) at a frequency of 100 kHz for 30 min to ensure uniformity. All the other chemicals used were of analytical grade and purchased from Himedia (Thane, India).

### Selection of test concentration and grouping

The test concentration was selected according to IONP dispersion, as mentioned in our previous publication (27). Following the acclimatisation phase, 160 fish were randomly distributed into eight 40-litre glass tanks (30 cm width, 60 cm length, and 30 cm depth), each holding 20 fish. The control group was not exposed to IONPs. Treatment groups were exposed to IONPs at 15 mg/L for 1, 3, 4, 15, 30, and 60 days (IONP1, 3, 4, 15, 30, and 60, respectively). The depuration group was first exposed to IONPs (15 mg/L) for 60 days, and then followed up in IONP-free water for another 60 days.

### Determination of the bioaccumulation factor (BAF)

IONP tissue accumulation was analysed in fish groups treated for four days (IONP4), 60 days (IONP60), and in the 60-day depuration group using a method described by Arslan et al. (28) with slight modifications. Fish from each group were captured with a small dip net to avoid additional stress, euthanised, and dissected. Gill, liver, and brain were excised and rinsed with cold saline before weighing. Wet tissue samples were weighed and digested with a mixture of concentrated nitric and hydrochloric acid (3:1) at 200 °C for 1 h, and the digested material was evaporated to remove any contaminants or residues that might interfere with analytical measurements. The remaining tissue was diluted with deionised water to a known volume. Total iron content, consisting of both iron nanoparticles and any other forms of iron within the samples, was quantified with inductively coupled plasma mass spectrometry (ICP-MS; Thermo Fisher, Waltham, MA, USA). The IONP content in total iron concentration was estimated based on the known molar mass of Fe_3_O_4_ and atomic mass, as follows:

[1] $${\mathrm{Fe}}_{3}O_{4}\;\mathrm{mass}=\frac{\mathrm{Fe}\;\mathrm{concentration}\times {\mathrm{Fe}}_{3}O_{4}\;\mathrm{molar}\;\mathrm{mass}}{\mathrm{Fe}\;\mathrm{atomic}\;\mathrm{mass}}$$

This conversion allowed us to determine the number of nanoparticles corresponding to the measured mass, which was used to calculate the bioaccumulation factor (BAF), expressed as micrograms per gram of wet tissue weight.

[2] $$\text{BAF}=\frac{\text{IONP}\;\text{concentration}\;\text{in}\;\text{tissue}\;\left(\mu \text{g}/\text{g}\right)}{\text{IONP}\;\text{concentration}\;\text{in}\;\text{water}\;\left(\text{mg}/\text{L}\right)}$$

### Preparation of tissue samples for biochemical analysis

At the end of every treatment, fish were captured as described above, weighed, euthanised, and dissected to remove the gill, liver, and brain. The tissues were then rinsed with cold saline and weighed. The hepatosomatic index (HSI) of the fish was calculated using the following equation:

[3] $$\text{HSI}=\frac{\text{Liver}\;\text{weight}\;\left(\text{mg}\right)}{\text{Total}\;\text{body}\;\text{weight}\;\left(\text{mg}\right)}\times 100$$

Tissue homogenates (1 % w/v) were prepared separately in normal saline on ice using a motor-driven tissue homogeniser (Remi, Mumbai, India) and the homogenates centrifuged at 800 *g* for 15 min at 4 °C to collect supernatants used for biochemical analyses. Total protein was estimated using bovine serum albumin (BSA) for the standard as described elsewhere (29). Activities of superoxide dismutase (SOD), catalase (CAT), glutathione reductase (GR), and glutathione peroxidase (GPx) were analysed in the gill, liver, and brain tissues. Activities of marker enzymes alkaline phosphatase (ALP) in the gill and liver and acetylcholinesterase (AChE) in the brain were estimated as described below.

### Superoxide dismutase (SOD) assay

SOD activity was determined as described by Marklund and Marklund (30). The assay mixture contained tris-hydrochloric acid buffer (50 mmol/L, pH 7.6) consisting of 1 mmol/L EDTA, 0.2 mmol/L pyrogallol, and 100 µL of enzyme source. The increase in absorbance was measured at 420 nm against an enzyme-free blank at 10-second intervals over 3 min using an ultraviolet (UV)-visible spectrophotometer (Shimadzu, Kyoto, Japan). Enzyme activity is expressed as nmol of oxidised pyrogallol per min per mg of protein.

### Catalase (CAT) assay

CAT activity was determined using the method described by Claiborne (31). A total mixture of 3 mL was composed of phosphate buffer (50 mmol/L, pH 7.0), hydrogen peroxide (19 mmol/L), and 50 µL of enzyme source. The decrease in absorbance was measured at 240 nm against an enzyme-free blank at 10-second intervals over 3 min using a UV-visible spectrophotometer (Shimadzu, Kyoto, Japan). Enzyme activity was expressed as mol of hydrogen peroxide consumed per min per mg of protein.

### Glutathione reductase (GR) assay

The activity of GR was assayed as described by Carlberg and Mannervik (32). The assay mixture comprised phosphate buffer (100 mmol/L, pH 7.6), NADPH (0.2 µmol/L), oxidised glutathione (2 mmol/L), EDTA (0.01 mol/L), and the enzyme source. The reduction of NADPH was monitored by measuring the decrease in absorbance at 340 nm against an enzyme-free blank at 10-second intervals over 3 min using a UV-visible spectrophotometer (Shimadzu). Enzyme activity is expressed as nmol of oxidised NADPH per min per mg of protein.

### Glutathione peroxidase (GPx) assay

The activity of GPx was determined with the method described by Mohandas et al. (33) utilising hydrogen peroxide and NADPH as substrates. The assay mixture comprised phosphate buffer (100 mmol/L, pH 7.6), EDTA (0.01 mol/L), sodium azide, GR, reduced glutathione, and NADPH (0.2 µmol/L). Enzyme assay was added and NADPH reduction monitored by measuring the decrease in its absorbance at 340 nm against an enzyme-free blank at 10-second intervals over 3 min using a UV-visible spectrophotometer (Shimadzu). Enzyme activity is expressed as nmol of oxidised NADPH per min per mg of protein.

### Quantification of hydrogen peroxide

The levels of hydrogen peroxide were assessed following the method described by Pick and Keisari (34). The assay is based on the H_2_O_2_-mediated and horseradish peroxidase-dependent oxidation of phenol red to a product. The incubation mixture comprised phosphate buffer (50 mmol/L, pH 7.6), horseradish peroxidase (8.5 units), phenol red (0.28 nmol/L), dextrose (5.5 nmol/L), and enzyme source (100 µL). The reaction was carried out at room temperature for 30 min, after which it was terminated by adding 10 eq/L sodium hydroxide. The absorbance was measured at 610 nm against the blank. A standard curve was prepared using known concentrations of H_2_O_2_, and the results are expressed as nmol of generated hydrogen peroxide per mg of protein.

### Lipid peroxidation (LPO) assay

LPO was measured using thiobarbituric acid as outlined by Ohkawa et al. (35). A working solution was prepared by combining 15 % w/v trichloroacetic acid, 0.37 % 2-thiobarbituric acid, and 0.25 eq/L hydrochloric acid in a 1:1:1 ratio. The enzyme source was added to the working solution at a ratio of 1:2 and incubated in a boiling water bath for 10 min. Followed the measurement of absorbance at 535 nm against the blank. Malondialdehyde (MDA) solution served as the standard, and the results are expressed as nmol of MDA per mg of protein.

### Alkaline phosphatase (ALP) assay

The activity of ALP in gill and liver tissues was determined following the method described by Bessey et al. (36). Pre-incubation involved *p*-nitrophenyl phosphate and glycine buffer at pH 10.5, maintained at 37 °C for 5 min. Subsequently, the sample and 0.02 eq/L NaOH were added to the mixture and incubated for 30 min. The resultant colour was measured at 420 nm using a UV-visible spectrophotometer (Shimadzu) against the blank. Upon the addition of 0.1 mL of concentrated hydrochloric acid, the mixture was thoroughly mixed and the difference in absorbance recorded as the measure of enzyme activity. The activity of ALP was determined from the calibration curve generated using the *p*-nitrophenol standard. Enzyme activity is expressed as µmol of liberated *p*-nitrophenol per min per mg of protein.

### Acetylcholinesterase (AChE) assay

The activity of AChE in the brain tissue was determined as described by Ellman et al. (37) by monitoring the increase in yellow colour resulting from the reaction between thiocholine and dithiobisnitrobenzoate (DTNB; 0.01 mol/L). Brain tissue was homogenised and dissolved in phosphate buffer (0.1 mol/L, pH 8.0), 15 mg of sodium bicarbonate, and DTNB. Enzyme activity is expressed as nmol of hydrolysed acetylthiocholine per min per mg of protein.

### Statistical analyses

Statistical analyses were run on the IBM SPSS Statistics version 21.0 for Windows (SPSS Inc., Chicago, MI, USA). Differences between the means were determined with one-way analysis of variance (ANOVA), followed by Duncan’s multiple range *post-hoc* test. Normality of distribution was tested with the Kolmogorov-Smirnov test and the homogeneity of variance between subsets analysed with Levene’s test. The significance was set at P<0.05 vs control. Data are presented as means ± standard deviations (SD) for 20 animals per group.

## RESULTS

### Characterisation of iron oxide nanoparticles

**Figure 1:**
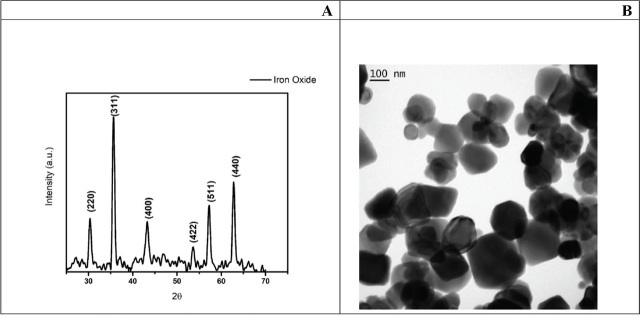
X-ray diffraction (XRD) image showing XRD peaks corresponding to IONPs with a particle size of 15.65 nm (A) and the transmission electron microscopy (TEM) image showing the morphology of IONP aggregates dispersed in double distilled water. Scale bar = 100 nm

The XRD results confirmed that IONPs used in our study were free from impurities. TEM analysis revealed a crystalline structure, with irregular and roughly symmetrical morphology, ranging in size from 1 to 100 nm. The average crystallite size calculated with the Scherrer equation was 15.65 nm. These results corroborate the manufacturer’s specifications (Figure 1).

### Body and tissue weights

Fish exposed to IONPs did not exhibit significant changes in body weight. However, gill weight increased while the hepatosomatic index decreased significantly (P<0.05) after 30 and 60 days of exposure (Table 1). Brain weight remained unchanged throughout the experiment. The hepatosomatic index returned to control levels in the depuration group, while body and organ weights remained unchanged (Table 1).

**Table 1:** The Effect of iron oxide nanoparticles (IONPs; 15 mg/L) on mean (±SD) body and tissue weights of the fish Mozambique tilapia (*Oreochromis mossambicus,* Peters 1852) by groups (N=20 each)

**Parameters**	**IONPs (15 mg/L)**							**Depuration group (60 days)**
	**Control**	**1 day**	**3 days**	**4 days**	**15 days**	**30 days**	**60 days**	
**Body weight (g)**	6.71±0.09	6.25±0.40	6.31±0.35	6.51±0.26	6.59±0.38	6.47±0.38	6.63±0.40	6.99±0.13
**Gill weight (mg)**	143±11.9	146±3.25	152±3.88	152±1.19	159±3.20	161±2.98*	172±1.41*	186±1.50*
**Hepatosomatic index (%)**	13.53±1.86	13.78±1.75	13.22±1.06	12.06±1.42	11.98±1.53	11.90±1.75*	9.84±1.26*	13.22±0.48
**Brain weight (mg)**	16.5±1.81	16.6±2.23	16.3±3.56	15.2±1.61	15.1±0.90	15.3±2.83	15.0±1.66	19.5±1.08

* P<0.05 compared to the control group

### Bioaccumulation of nanoparticles in tissues

The ICP-MS analysis confirms the presence of IONPs in the gill, liver, and brain tissues of the fish after 4 and 60 days of exposure (Table 2). The gill tissue exhibited the highest bioaccumulation factor, followed by the liver and brain. The 60-day depuration failed to restore IONP levels to control.

**Table 2:** Mean (±SD) Bioaccumulation of iron oxide nanoparticles (IONPs; 15 mg/L) in the gill, liver, and brain tissues of the fish Mozambique tilapia (*Oreochromis mossambicus,* Peters 1852) exposed for 4 and 60 days, and fish followed up for 60 days after 60-day exposure had stopped (depuration period) (N=20 per group)

**Concentration of IONPs in water (mg/L)**	**Exposure duration**	**Tissues**	**IONP tissue concentrations (µg/mg)**	**BAF**
**0 (Control)**	60 days	Gill, liver, and brain	Below detection limit	Below detection limit
**15**	4 days	Gill	0.53±.017	0.04
		Liver	0.19±0.01	0.01
		Brain	0.15±0.01	0.01
	60 days	Gill	7.87±0.26	0.53
		Liver	6.25±0.13	0.42
		Brain	1.86±0.10	0.13
	Depuration (60 days)	Gill	2.20±0.08	0.15
		Liver	1.90±0.08	0.13
		Brain	0.92±0.13	0.06

BAF – bioaccumulation factor

### Biochemical analysis

**Figure 2:**
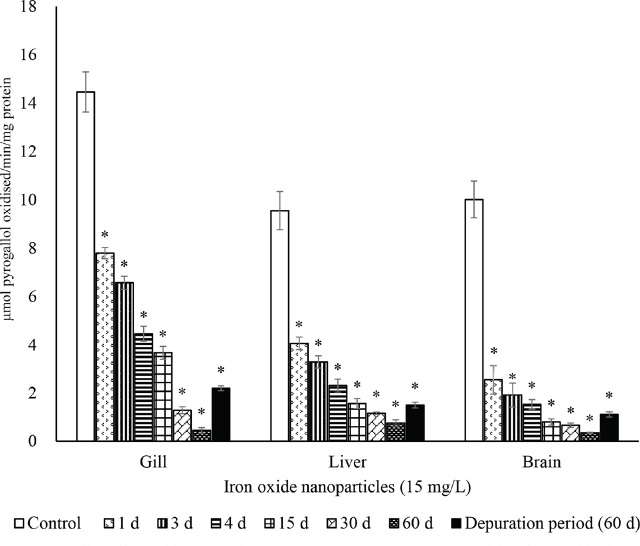
The effect of iron oxide nanoparticles (IONPs; 15 mg/L) on mean (±SD) activity of superoxide dismutase (SOD) in the fish Mozambique tilapia (*Oreochromis mossambicus,* Peters 1852) by groups (N=20 each). *P<0.05 vs control

**Figure 3:**
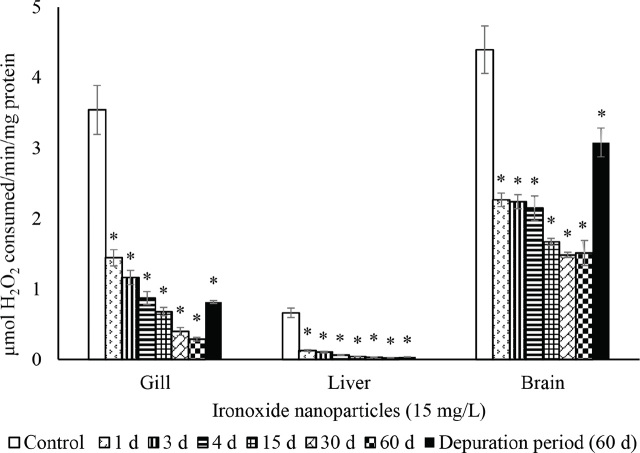
The effect of iron oxide nanoparticles (IONPs; 15 mg/L) on mean (±SD) activity of catalase (CAT) in the fish Mozambique tilapia (*Oreochromis mossambicus,* Peters 1852) by groups (N=20 each). *P<0.05 vs control

**Figure 4:**
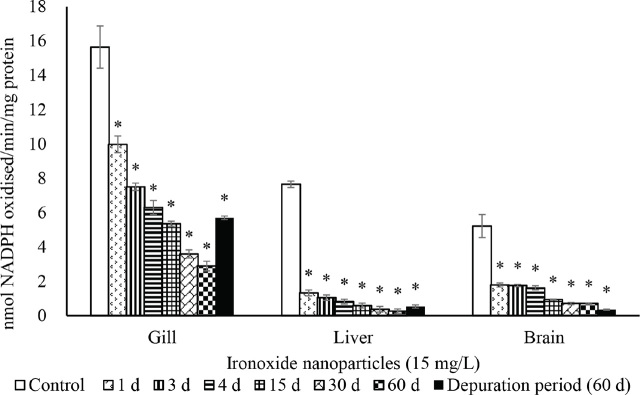
The effect of iron oxide nanoparticles (IONPs; 15 mg/L) on mean (±SD) activity of glutathione reductase (GR) in the fish Mozambique tilapia (*Oreochromis mossambicus,* Peters 1852) by groups (N=20 each). *P<0.05 vs control

**Figure 5:**
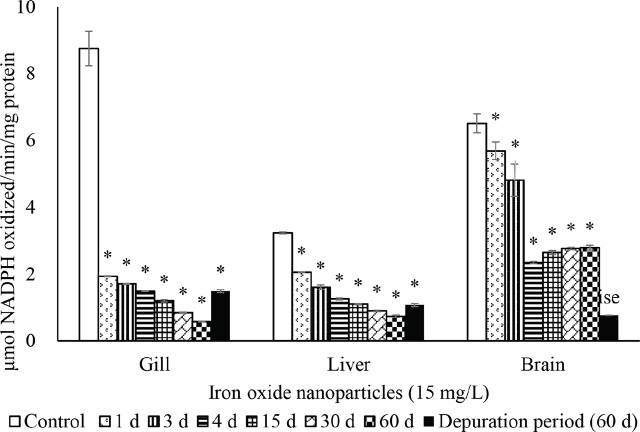
The effect of iron oxide nanoparticles (IONPs; 15 mg/L) on mean (±SD) activity of glutathione peroxidase (GPO) in the fish Mozambique tilapia (*Oreochromis mossambicus,* Peters 1852) by groups (N=20 each). *P<0.05 vs control

**Figure 6:**
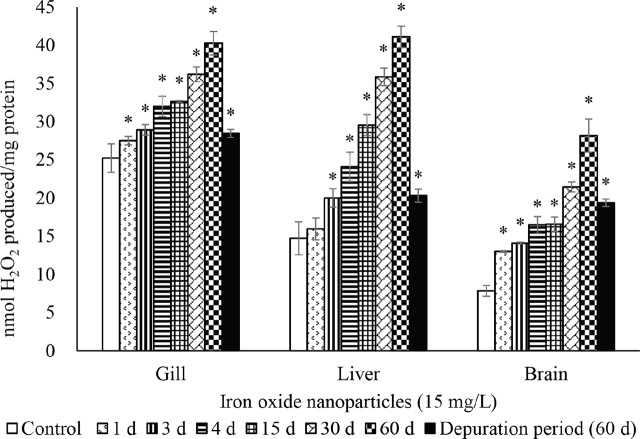
The effect of iron oxide nanoparticles (IONPs; 15 mg/L) on mean (±SD) mean (±SD) hydrogen peroxide levels in the fish Mozambique tilapia (*Oreochromis mossambicus,* Peters 1852) by groups (N=20 each). *P<0.05 vs control

**Figure 7:**
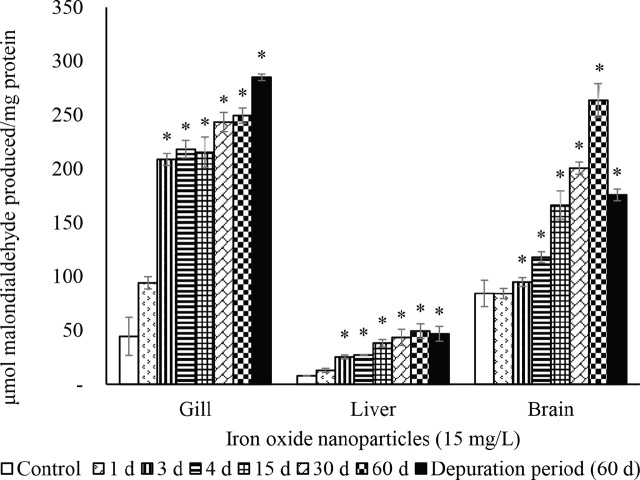
The effect of iron oxide nanoparticles (IONPs; 15 mg/L) on mean (±SD) lipid peroxidation (LPO) in the fish Mozambique tilapia (*Oreochromis mossambicus,* Peters 1852) by groups (N=20 each). *P<0.05 vs control

The activities of SOD (Figure 2), CAT (Figure 3), GR (Figure 4), and GPx (Figure 5) showed a significant (P<0.05) decrease in the gill, liver, and brain in a time-dependent manner compared to respective controls. Conversely, the levels of hydrogen peroxide (Figure 6) and LPO (Figure 7) increased significantly (P<0.05) in all tissues in a time-dependent manner. Upon 60-day depuration, these levels did not return to control (Figures 2–7).

**Figure 8:**
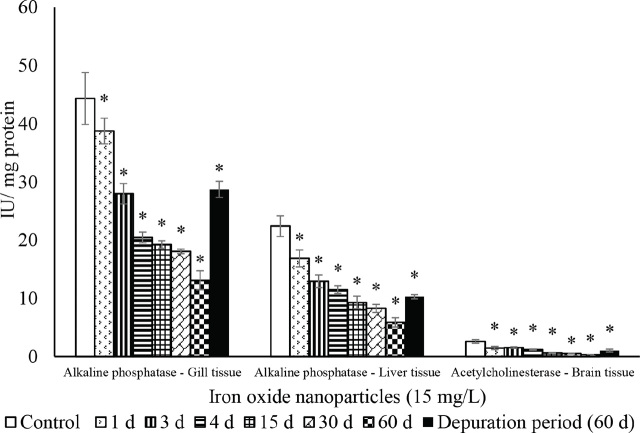
The effect of iron oxide nanoparticles (IONPs; 15 mg/L) on mean (±SD) activity of alkaline phosphatase (ALP) in the fish Mozambique tilapia (*Oreochromis mossambicus,* Peters 1852) by groups (N=20 each). *P<0.05 vs control

In both gill and liver, ALP activity dropped significantly (P<0.05) in a time-dependent manner. A similar significant and time-dependent drop was observed for brain tissue AChE activity. Again, 60-day depuration did not reverse the activities of these enzymes (Figure 8).

## DISCUSSION

The accumulation of the IONPs in the gill, liver, and brain tissues of *Oreochromis mossambicus* determined in our study underscores their ability to easily enter the organism, accumulate in vital organs, and potentially alter their functions (38, 39). As reported by Ates et al. (40), who studied chronic exposure of tilapia (*Oreochromis niloticus*) to IONPs, the uptake, assimilation, and immunotoxic effect much depend on the morphological properties of IONPs. Small nanoparticle size facilitates penetration into organisms and subsequent accumulation in tissues, a phenomenon that has less been explored in aquatic bioaccumulation studies. It is crucial to understand whether nanoparticles directly incorporate into tissues, cells, and biomolecules, leading to toxicological responses, or undergo chemical changes and transformations that indirectly contribute to toxicity. In a previous study, we also reported histopathological changes induced by IONPs in these tissues of *Oreochromis mossambicus* (26). Our findings align with those of Murali et al. (41) on the accumulation of three different concentrations of aluminium oxide nanoparticles in the liver tissue resulting in severe histopathological changes.

Like all aerobic organisms, fish have well-defined and functional prooxidant/antioxidant systems to counter oxidative stress and the formation of reactive oxygen species (ROS) (42). In our study, exposure to IONPs resulted in depletion of antioxidant enzymes such as SOD, CAT, GR, and GPx in all tissues. This depletion resulted in the accumulation of free radicals, as evidenced by increased levels of hydrogen peroxide. Similar results have been observed in the gill and liver tissues of freshwater orange chromide (*Pseudetroplus maculatus*) upon exposure to fullerene C_60_ for 96 h (43, 44). Furthermore, the 60-day recovery from nanoparticles exposure (depuration period) did not significantly improve antioxidant enzyme activity or restore it to control levels, which suggests that these effects of nanotoxicity may be long-term. They may be owed to changes in vital protein conformations (45), irreversible cytoskeleton damage (46), or induced adsorption of nanoparticles by lipid bilayers as a result of lipophilicity of cationic ligands (47).

Nanoparticle toxicity is owed to oxygen depletion and accumulation of free radicals (48, 49). Lipids, polyunsaturated fatty acids (PUFA) in particular, possess several double bonds and are highly susceptible to free radical attacks. Exposure to IONPs for 60 days resulted in a significant rise in LPO levels in all three tissues of our test organism, which suggests that gill, liver, and brain are equally targeted by oxidative stress and corroborates our earlier findings of elevated LPO levels in the liver of the same species (50).

Considering that ALP is involved in the hydrolysis of exogenous materials, transphosphorylation, and membrane transport (51), its decreased activity in the gill and liver tissues points to impaired membrane transport and cellular toxicity (50).

The activity of the brain tissue marker, AChE, the key enzyme involved in the breakdown of the neurotransmitter acetylcholine to terminate synaptic neurotransmission (51), dropped in a time-dependent manner, indicating the neurotoxic effects of IONPs. The activities of these marker enzymes remained depleted throughout the depuration period, which corroborates long-term nanotoxic effects.

## CONCLUSION

Clearly, oxidative stress plays a major role in nanoparticle-induced toxicity, and nanoparticles execute it through different mechanisms like generating ROS, disrupting cellular antioxidant defence system, interacting and damaging biomolecules like proteins, lipids, and DNA, as well as by disrupting membrane integrity (52,53,54,55). Our findings well illustrate the cascade of events and the relation between different mechanisms that could lead to oxidative stress. IONP accumulation in the gill, liver, and even brain tissue confirms their internalisation by fish and also the ability to cross the blood-brain barrier and induce neurotoxicity. Accumulation of IONPs inside vital organs disrupted the antioxidant defence system by depleting antioxidant enzymes, resulting in redox imbalance, which in turn induced LPO and likely impaired cell membrane integrity.

Our study also highlights the persistence of IONPs and their toxic effects in the fish even after the depuration period. We therefore need to consider long-term exposure to IONPs and their exacerbating toxicity over time, as it has various biological implications for humans. Even though human health risks resulting from occupational and environmental exposure to IONPs are negligible, *in vitro* studies on human cells such as blood (56), endothelial (57, 58), neuroblastoma and glioblastoma (59), alveolar epithelial cells (60), neutrophils (61), and astrocytes (62) point to the potential risks of IONP exposure and the need for caution before applying IONPs in clinical settings. However, further studies are warranted to shed more light on the nature IONP toxicity and its implications for humans.

Furthermore, our findings highlight the need for further investigations into the potential risks associated with nanoparticle exposure in aquatic environments.
